# Discovery of a small-molecule NDR1 agonist for prostate cancer therapy

**DOI:** 10.3389/fphar.2024.1367358

**Published:** 2024-02-12

**Authors:** Yang Bai, Xiuyuan Sui, Zuodong Xuan, Yifan Du, Meiling Fu, Zeyuan Zheng, Kunao Yang, Chunlan Xu, Yankuo Liu, Bin Liu, Min Zhong, Zhengying Zhang, Jianzhong Zheng, Xiaoyan Hu, Lei Zhang, Huimin Sun, Chen Shao

**Affiliations:** ^1^ School of Medicine, Xiamen University, Xiamen, Fujian, China; ^2^ Department of Urology, Xiang’an Hospital of Xiamen University, School of Medicine, Xiamen University, Xiamen, Fujian, China; ^3^ School of Public Health, Xiamen University, Xiamen, Fujian, China

**Keywords:** NDR1, castration-resistant prostate cancer, small-molecule compound, cancer therapy, target

## Abstract

Prostatic cancer (PCa) is a common malignant neoplasm in men worldwide. Most patients develop castration-resistant prostate cancer (CRPC) after treatment with androgen deprivation therapy (ADT), usually resulting in death. Therefore, investigating new therapeutic targets and drugs for PCa patients is urgently needed. Nuclear Dbf2-related kinase 1 (NDR1), also known as STK38, is a serine/threonine kinase in the NDR/LATS kinase family that plays a critical role in cellular processes, including immunity, inflammation, metastasis, and tumorigenesis. It was reported that NDR1 inhibited the metastasis of prostate cancer cells by suppressing epithelial-mesenchymal transition (EMT), and decreased NDR1 expression might lead to a poorer prognosis, suggesting the enormous potential of NDR1 in antitumorigenesis. In this study, we characterized a small-molecule agonist named aNDR1, which specifically bound to NDR1 and potently promoted NDR1 expression, enzymatic activity and phosphorylation. aNDR1 exhibited drug-like properties, such as favorable stability, plasma protein binding capacity, cell membrane permeability, and PCa cell-specific inhibition, while having no obvious effect on normal prostate cells. Meanwhile, aNDR1 exhibited good antitumor activity both *in vitro* and *in vivo*. aNDR1 inhibited proliferation and migration of PCa cells and promoted apoptosis of PCa cells *in vitro*. We further found that aNDR1 inhibited subcutaneous tumors and lung metastatic nodules *in vivo*, with no obvious toxicity to the body. In summary, our study presents a potential small-molecule lead compound that targets NDR1 for clinical therapy of PCa patients.

## 1 Introduction

Prostatic cancer (PCa) is one of the most common urogenital malignant cancers, ranking second in incidence and fifth in mortality among men worldwide (Sung et al., 2021). Initially, most prostate cancer patients respond well to androgen deprivation therapy (ADT), including surgical and medical castration therapy (Le et al., 2023). However, after several years, the majority of patients become insensitive to these therapeutic regimens and develop castration-resistant prostate cancer (CRPC), which often leads to death (Zhong et al., 2021a; Zhong et al., 2021b). Although many treatment options have been developed, including endocrinotherapy, chemotherapy and immunotherapy, the efficacy of these treatments against CRPC is enormously restricted because of drug resistance and individual variation (Cai et al., 2023). Therefore, searching for new therapeutic targets and drugs as alternative treatment options for PCa patients is crucial.

NDR1, also known as STK38, belongs to the NDR (nuclear Dbf2 related) kinase family, which has been found in various species, including yeast, fruit flies, and mammals (Ye et al., 2020), regulating cell mitosis, embryonic development, centrosome replication and organ size (Johnston et al., 1990). Currently, it is believed that NDR1 mainly functions as a member of the Hippo pathway by cooperating with NDR2, LATS1 and LATS2 to phosphorylate YAP and regulate its activity (Hergovich, 2016; Liu et al., 2016; Hong et al., 2018). NDR1 inhibits the progression of colorectal cancer, T-cell lymphoma, glioblastoma and prostate cancer (Cornils et al., 2010; Zhang et al., 2015; Yue et al., 2018; Chen et al., 2021; Khan et al., 2023), also plays an important role in infection, inflammation and immunity (Shi et al., 2012; Wen et al., 2015; Ma et al., 2017; Ye et al., 2020) and promotes apoptosis and autophagy (Vichalkovski et al., 2008a; Joffre et al., 2015). Our previous work has shown that NDR1 inhibits the metastasis of prostate cancer cells by suppressing epithelial-mesenchymal transition (EMT), and decreased NDR1 expression might lead to a poorer prognosis in patients (Yue et al., 2018). Considering the critical functions of NDR1, the pharmacological activation of NDR1 may play a significant role in tumor therapy. However, no agonist targeting NDR1 has been investigated. Therefore, the development of a small-molecule agonist of NDR1 is urgently needed.

In this study, we used the FTMAP service and ChEMBL database to identify a small-molecule lead compound named aNDR1, and we found that it specifically binds to NDR1 and potently promotes NDR1 expression, enzymatic activity and phosphorylation. aNDR1 has favorable physicochemical properties, such as chemical structure stability, plasma protein binding capacity and cell membrane permeability. Our study found that aNDR1 effectively inhibited proliferation and migration and promoted apoptosis of prostate cancer cells *in vitro*, and knockdown of NDR1 prevented these effects. Meanwhile, aNDR1 inhibited subcutaneous tumors and lung metastatic nodules *in vivo*, with no obvious toxicity to the body.

## 2 Materials and methods

### 2.1 Screening, synthesis and verification of aNDR1

aNDR1 was screened and identified as an agonist of NDR1. The X-ray crystal structure of NDR1 (code: 6BXI) was downloaded from the RCSB PDB database (http://www.rcsb.org). FTMAP (http://ftmap.bu.edu/login.php) was used to identify potential binding sites for ligands by utilizing small-molecule probes. The contact frequency per residue with probes was generated to quantify the contribution of each residue to ligand binding. The results of FTMAP were visualized using PyMOL to generate an isopotential diagram depicting the molecular pocket (Seeliger and de Groot, 2010). The synthetic protocol of aNDR1 is shown in Supplementary Figure S1A, and its structure was verified through LC‒MS and ^1^H NMR. The purity of aNDR1 was greater than 95%, and it was dissolved in DMSO and stored at −20 °C.

### 2.2 Cell lines and culture conditions

Human embryonic kidney cells (HEK293T), WPMY-1 cells, and human prostate cancer cell lines (PC3 and DU145) were purchased from the cell bank of the Chinese Academy of Sciences (Shanghai, China). The cells were cultured in RPMI-1640 or DMEM containing 10% fetal bovine serum (FBS) and 1% penicillin‒streptomycin in a humidified 5% CO_2_ atmosphere at 37 °C.

### 2.3 Cell viability assay

The PC3, DU145 and WPMY-1 cells were seeded in 96-well plates at 5 × 10^3^ cells per well. Following 24 h of incubation, the cells were treated with different concentrations of aNDR1 for 24 h, followed by the addition of 10 µL CCK-8 to each well for 4 h. The absorbance was measured at 450 nm using a microplate reader. Cell viability (%) = OD (treated)/OD (control) × 100%.

### 2.4 Measurement of the stability, protein binding activity and cell permeability of aNDR1 in cell culture medium

aNDR1 was added to the cell culture medium at a concentration of 5 μM. The samples were obtained at 0, 24, 48, and 72 h and analyzed by UPLC-HRMS (Thermo Fisher Scientific, United States) to measure the stability of aNDR1. For protein binding activity, the medium with 10% FBS and 5 μM aNDR1 was centrifuged (230,000 g) for 5 h at 37 °C. The supernatant was analyzed by UPLC‒HRMS (Thermo Fisher Scientific, United States) to detect unbound aNDR1. The permeability experiment model of Caco-2 cells was established according to a previous study (Xiao et al., 2022). aNDR1 was added to the apical compartment at a final concentration of 5 μM at 37 °C. Samples were taken from the compartments 2 h before and after incubation and then analyzed by UPLC‒HRMS (Thermo Fisher Scientific, United States).

### 2.5 Plasmid transfection and lentivirus transduction

In short, pMD2.0G and psPAX were cotransfected with sh-NDR1 or control vectors into HEK293T cells using Lipofectamine 3,000 (Invitrogen, United States) according to the manufacturer’s protocol. The supernatant containing lentivirus was collected after 48 h and subjected to infection of DU145 cells to construct NDR1-knockdown cells. Similarly, PCDNA3.1 and NDR1 plasmids were transfected by using Lipofectamine 3,000 (Invitrogen, United States) to overexpress NDR1 in HEK293T cells.

### 2.6 Bacterial expression and purification of human GST-fused NDR1 and the kinase activity assay

*E. coli BL21* was transformed with the pGEX-GST-NDR1 plasmid. Mid-logarithmic phase cells were induced with 1 mM isopropyl-β-D- thiogalactopyranoside (IPTG) overnight at 16 °C. The bacteria were collected and lysed in lysis buffer (20 mM Tris-Cl, pH 8.0, 100 mM NaCl, 0.2 mM EDTA, and 0.5% NP-40) together with lysozyme and phenylmethylsulfonyl fluoride (PMSF) via an ultrasonic cell crusher. Then, the GST-fused NDR1 proteins in the supernatant were purified on glutathione-agarose (GE Healthcare, United States) and detected by SDS‒PAGE-coupled Coomassie blue staining. According to the manufacturer’s protocol, purified NDR1 proteins were incubated with substrate peptide (KKRNRRLSVA), ATP, reaction buffer and different concentrations aNDR1 and the activity of NDR1 kinase was detected by a Kinase-Lumi™ luminescent kinase assay kit (Beyotime, China).

### 2.7 EdU proliferation assay

The proliferation of PCa cells was measured using the EdU Cell Proliferation Assay Kit (Beyotime, China). DU145 and PC3 cells were treated with aNDR1 or DMSO, stained with 5-ethynyl-20-deoxyuridine (EdU) according to the manufacturer’s instructions and photographed under a fluorescence microscope.

### 2.8 Wound healing assay

PC3 and DU145 cells were seeded in 6-well plates. At 95% confluence, a pipette was used to draw a straight line per plate, and PBS was used to remove cell debris. Photos were taken to record the beginning of wound gaps, and then medium containing aNDR1 or DMSO was added. The gap growth of each group was observed and photographed after 24 h. The wound healing rate was calculated by the scratch length change = (the average wound length at 0 h - the average wound length at 24 h)/the average wound length at 0 h.

### 2.9 Cell apoptosis and flow cytometry analysis

PC3 and DU145 cells were seeded in 6-well plates to a certain number, and then aNDR1 was added to the medium for 24 h. Next, all adherent and suspended cells were collected, and an Annexin V-FITC apoptosis detection kit (Beyotime Biotechnology, China) was used according to the manufacturer’s protocol. Apoptosis of cultured cells was detected via flow cytometry.

### 2.10 Cellular thermal shift assay (CETSA)

DU145 cells were transfected with MYC-NDR1 and treated with or without aNDR1 for 24 h. Cells were collected, and equal volumes of cell suspensions were heated at different temperatures for 5 min. Then, the cells were lysed by three freezes‒thaw cycles in liquid nitrogen, and the supernatant was collected after centrifuging the lysates at 15,000 × *g* for 15 min at 4 °C. The samples were detected via Western blotting analysis.

### 2.11 Drug affinity responsive target stability (DARTS)

After transfecting DU145 cells with MYC-NDR1 for 24 h, cells were lysed, and the supernatant was collected after centrifuging lysates at 15,000 × *g* for 15 min at 4 °C. Each sample was incubated with aNDR1 for 1 h at room temperature. Then, different concentrations of pronase were added to lysates and digested for 5 min. Lysates were added to loading buffer and then subjected to boiling and Western blotting analysis.

### 2.12 Western blot analysis and immunoprecipitation

Cells were lysed with RIPA lysis buffer (Solarbio, China) containing protease and phosphatase inhibitors (MedChemExpress, United States). An equal amount of protein in each sample was isolated by SDS‒PAGE electrophoresis, transferred to a PVDF membrane (Millipore, United States), and then blocked with 5% fat-free milk or 3% bovine serum albumin (BSA) in Tris-buffered saline with Tween 20 (TBST) at room temperature for 1 h. The membrane was then incubated with the primary antibody, and the general Western blotting assay procedure was followed. For immunoprecipitation, the starting steps were as described above but involved the use of IP lysate (Thermo Fisher Scientific, United States). A portion of the protein solution was taken to directly detect the corresponding protein expression, while the rest of the solution was added to the primary antibody and magnetic beads (Bimake, United States), respectively, according to the manufacturer’s instructions. The beads were washed with wash buffer (Thermo Fisher Scientific, United States) and heated to 100 °C for 5 min using protein loading buffer (ABclonal, China). Immunoblotting was then performed as described above.

### 2.13 Antibodies

The NDR1 (sc-365555) antibody was purchased from Santa Cruz Technology. The phospho-NDR1/2 (Thr444/Thr442) antibody was purchased from Affinity Biosciences. All the following antibodies were purchased from Cell Signaling Technology: E-cadherin (#14472), PARP (#9532), cleaved-PARP (#5625), phospho-Thr (#9386), β-actin mouse monoclonal antibody (#3700), and secondary antibody against mouse and rabbit. Bcl-2 (A19693), Bax (A19684), N-cadherin (A19083), Vimentin (A19607), Survivin (A1551), YAP (A1002), and phospho-YAP-Ser127 (AP0489) were purchased from ABclonal. All antibodies were used following the manufacturer’s instructions.

### 2.14 Animal studies

All wild-type male BALB/c nude mice were purchased from the Experimental Animal Center of Xiamen University (Xiamen, China). DU145 cells (1 × 10^6^) were injected subcutaneously into the backs of nude mice (6–8 weeks). When the tumor volume grew to 50–100 mm^3^, the mice were randomly divided into two groups: the control group and the aNDR1 treatment group. These two groups were treated with the same concentrations of placebo or aNDR1 (5 mg/kg) every other day. The body weight of the mice and the length and width of the tumor were measured every other day, and the tumor volume was calculated using Equation V= (length x width^2^)/2, where length was always the longest size of the tumor. At the end of the experiment, the mice were sacrificed, and the solid tumors were resected, weighed and sacrificed. The tumor tissues and important organs were analyzed by H&E staining. For the metastasis model, DU145 cells (1 × 10^6^) were intravenously injected into the tail veins of nude mice, and the mice were randomly divided into two groups: the control group and the aNDR1 treatment group. Similarly, these two groups were treated with the same concentrations of placebo or aNDR1 (5 mg/kg) every other day. After 14 days, the mice were sacrificed, and the number of lung metastasis colonies was counted.

### 2.15 Statistical analysis

GraphPad Prism 8 software was used to perform statistical analysis. All experiments were repeated at least three times except for the animal experiments. The significance of the differences between the two groups was assessed using Student’s t-test. Univariate and repeated measure analysis of variance (ANOVA) was used to compare the significance. ^*^ *p* < 0.05; ^**^ *p* < 0.01; ^***^ *p* < 0.001; NS, not significant.

## 3 Results

### 3.1 aNDR1 is screened as a small-molecule agonist of NDR1

**FIGURE 1:**
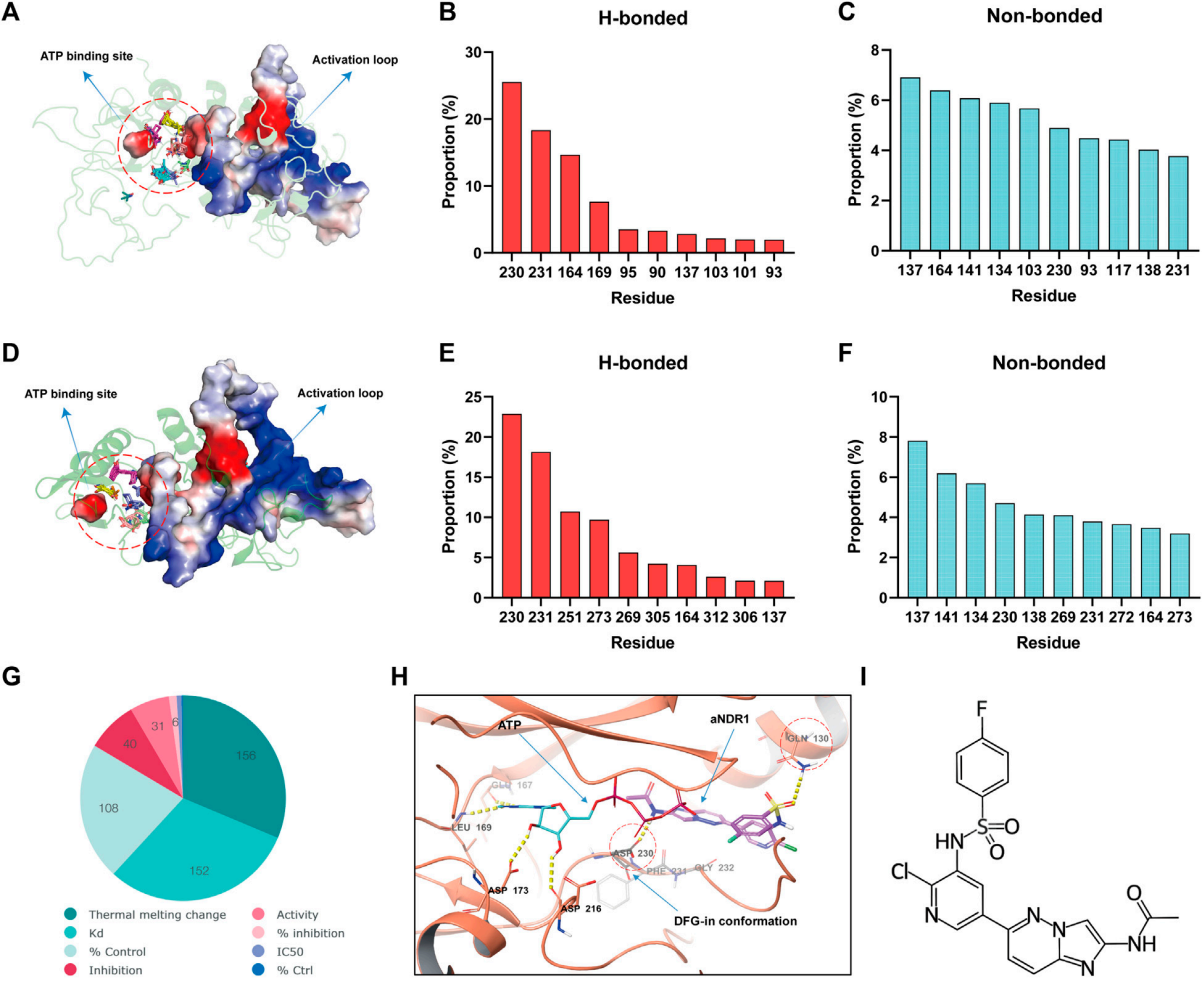
The small-molecule compound aNDR1 is a potential agonist of NDR1. **(A)** Probe distribution at the NDR1-full revealed by FTMAP. Probes were shown as lines. Red circle represents the space between activation loop and ATP binding site. **(B,C)** Proportions of top ten hydrogen-bond (h-bond, HB) **(B)** and non-bond **(C)** interaction for residues that made contact with probes used by FTMAP. **(D)** Probe distribution at the NDR1-protein kinase domain revealed by FTMAP. Probes were shown as lines. Red circle represents the space between activation loop and ATP binding site. **(E,F)** Proportions of top ten hydrogen-bond (h-bond, HB) **(E)** and non-bond **(F)** interaction for residues that made contact with probes used by FTMAP. **(G)** Small-molecule compounds target NDR1 in ChEMBL database. **(H)** Molecule docking among aNDR1, ATP and NDR1 protein. **(I)** The chemical structure of aNDR1.

Considering that NDR1 belongs to the NDR kinase family, the activation loop is very important for kinase activity. The activation loop usually begins with the Asp-Phe-Gly (DFG) motif and ends with the Ala-Pro-Glu (APE) motif. In the fully active state, the DFG motif adopts the so-called DFG-in state, wherein the Asp of DFG points inside the active site cleft (Xie et al., 2020). Many small-molecule drugs function by activating or inhibiting activation loops, especially the DFG motif. To identify a specific small molecule agonist of NDR1, we initially conducted druggability analysis of the NDR1-full and NDR1-protein kinase domain using FTMAP (Ngan et al., 2012). The analysis revealed that the activation loop region (from Asp230 to Glu292) was the most promising druggable pocket because of the high probe binding frequency in the NDR1-full (Figures 1A–C) and NDR1-protein kinase domain (Figures 1D–F). Subsequently, we focused on ChEMBL, a database of bioactive molecules with drug-like properties (Gaulton et al., 2012), to screen for small-molecule agonists of NDR1, and it provided 31 potential compounds that may activate NDR1 (Figure 1G). Then, we followed the principles of “Rule of 5" (Ro5) (Brown and Wobst, 2021). As a result, we obtained 19 potential small-molecule compounds (Supplementary Table S1).

To further evaluate the binding affinity and the most promising compound, we performed molecular docking (Pinzi and Rastelli, 2019) using computer software. Surprisingly, we found that the Compound C19H14ClFN6O3S (CHEMBL1822054, PubChem CID: 44124863) could form H-bonds with Asp230 Gln130, with a high docking score of −6.098 kcal/mol, which stabilized the DFG-in conformation and facilitated ATP binding, possibly promoting kinase activity (Figure 1H). We named this small-molecule compound aNDR1 (Figure 1I). Additionally, we synthesized aNDR1 (Supplementary Figure S1A) and verified its structure through ^1^H NMR (Supplementary Figure S1B) and LC‒MS (Supplementary Figure S1C).

### 3.2 aNDR1 specifically inhibits CRPC cells and promotes kinase activity of NDR1

**FIGURE 2:**
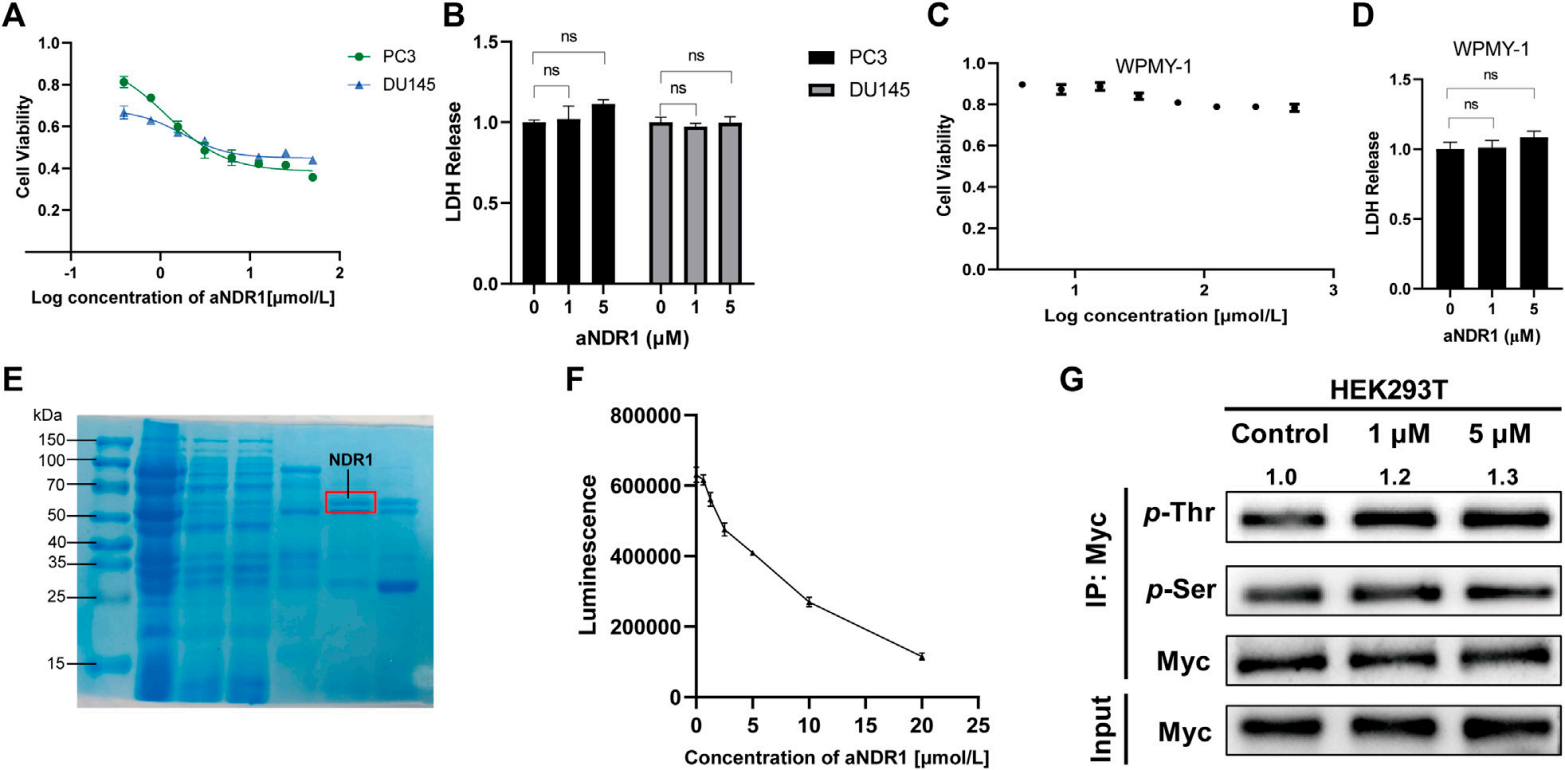
aNDR1 specifically inhibits CRPC cells and promotes kinase activity of NDR1. **(A)** The effect of aNDR1 on cell viability in prostate cancer cells PC3 and DU145 by CCK-8 assay. **(B)** The effect of aNDR1 on cell LDH release in prostate cancer cells PC3 and DU145. **(C)** The effect of aNDR1 on cell viability in normal prostate cells WPMY-1 by CCK-8 assay. **(D)** The effect of aNDR1 on cell LDH release in normal prostate cells WPMY-1. **(E)** Representative gel image stained with Coomassie Brilliant Blue of pull-down assay with GST-fused NDR1 in *Escherichia coli* BL-21 lysates. Red circle represents the NDR1 protein. **(F)** aNDR1 enhanced kinase activity of NDR1. The purified NDR1 protein from *Escherichia coli* was incubated with substrate peptide, ATP, reaction buffer and different concentrations aNDR1 for 30 min, then added to luciferin for 10 min. The luminescence intensity was detected by spectrophotometer to assess kinase activity. **(G)** aNDR1 increased phosphorylation of NDR1. Overexpressed Myc-NDR1 in HEK293T cells, treated with aNDR1 (0, 1, 5 μM), enriched NDR1 protein by IP and detected pan-phosphorylation of Thr and Ser. Data were represented as means ± SD of triplicate experiments. *, means *p* < 0.05, **, means *p* < 0.01, ***, means *p* < 0.001.

First, we evaluated the effects of aNDR1 in the CRPC cell lines PC3 and DU145. In cell viability assays, after 24 h of treatment with different concentrations of aNDR1, significant growth inhibition was observed in PCa cells. The IC_50_ values for PC3 and DU145 cells were determined to be 1.178 μM and 1.763 μM, respectively (Figure 2A). Subsequently, we assessed the cytotoxicity of aNDR1 in PCa cells using an LDH release assay. The results showed that aNDR1 had no obvious cytotoxicity (Figure 2B). To verify whether the effect of aNDR1 is tumor specific, we also performed similar experiments on the normal prostate cell line WPMY-1. The results showed that the IC_50_ of aNDR1 on WPMY-1 was greater than 500 μM (Figure 2C). Additionally, aNDR1 did not display noticeable cytotoxicity in WPMY-1 cells (Figure 2D). These findings suggest that aNDR1 selectively targets CRPC cells and its efficacy is not solely dependent on its own pharmacochemical properties.

NDR1 is known as an autophosphorylated kinase (Cook et al., 2014), which is fundamental for activity regulation. To eliminate the influence of autophosphorylation, we purified the NDR1 protein in *E. coli BL21* and confirmed it by Coomassie blue staining (Figure 2E). The kinase activity of NDR1 was assessed using a chemiluminescence kinase activity assay kit. The results indicated that NDR1 activity increased in a dose-dependent manner (Figure 2F). The Thr pan-phosphorylation of NDR1 was gradually elevated in response to the increased concentration of aNDR1, and Ser pan-phosphorylation had no obvious change (Figure 2G). These findings confirm that aNDR1 promotes NDR1 kinase activity.

Then, we investigated the physicochemical properties of aNDR1, including its chemical structure stability and plasma protein binding capacity as well as its cell membrane permeability by UPLC‒HRMS. The results revealed that aNDR1 maintained its structural integrity, with 84.93%, 83.80%, and 80.33% of the compound remaining intact after 1, 2, and 3 days of incubation in cell culture medium, respectively (Supplementary Figure S2A). In addition, 60.15% of aNDR1 was found to bind with proteins in the culture medium (Supplementary Figure S2B). Furthermore, aNDR1 exhibited efficient permeability across Caco-2 cells, with an apparent permeability coefficient (Papp) of 8.31 × 10^−6^ cm s^-1^ (Supplementary Figure S2C). These data suggest that aNDR1 possesses favorable drug-like physicochemical properties.

### 3.3 aNDR1 inhibits proliferation and migration and induces apoptosis of PCa cells *in vitro*

**FIGURE 3:**
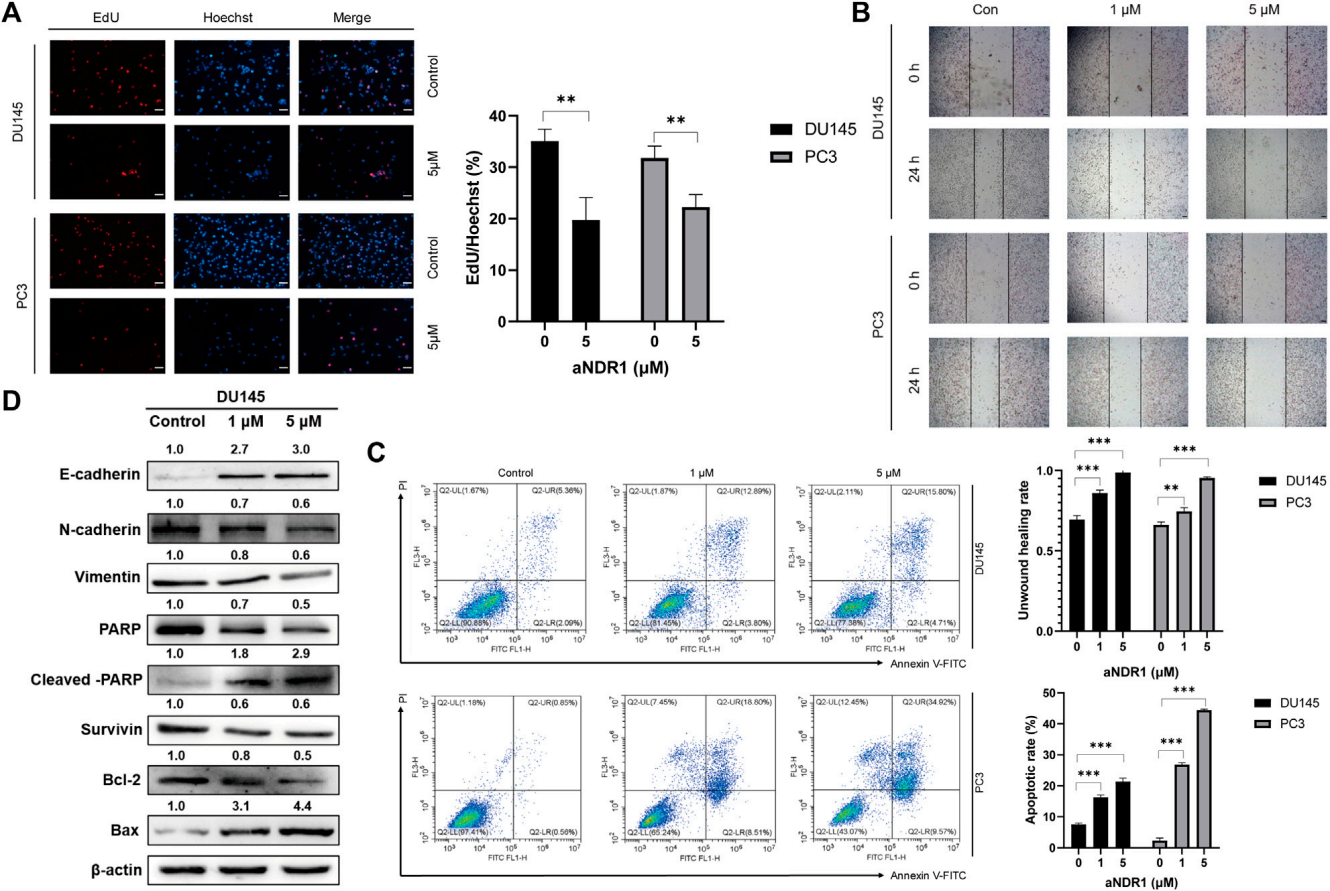
aNDR1 inhibits proliferation and migration, and induces apoptosis of PCa cells *in vitro* **(A)** aNDR1 inhibited the proliferation of PCa cells. DU145 and PC3 cells were treated with aNDR1 (0, 5 μM) for 24 h, and then stained by EdU and Hoechst. Scale bar, 50 μm. **(B)** aNDR1 inhibited the migration of PCa cells. DU145 and PC3 cells were treated with aNDR1 (0, 1, 5 μM) for 24 h, and cell migration was detected using a monolayer wound healing assay. Scale bar, 100 μm. **(C)** aNDR1 promoted the apoptosis of PCa cells. Analysis of apoptotic cells induced by aNDR1 for 24 h using flow cytometry. **(D)** aNDR1 inhibited EMT and promoted apoptosis. Western blot analysis of EMT and apoptosis related proteins after treatment with aNDR1 or DMSO. Data were represented as means ± SD of triplicate experiments. *, means *p* < 0.05, **, means *p* < 0.01, ***, means *p* < 0.001.

We further investigated the effects of aNDR1 on prostate cancer cells *in vitro*. PC3 and DU145 cells were exposed to varying concentrations of aNDR1 for 24 h, followed by labeling with EdU and Hoechst. As shown in Figure 3A, the proportion of EdU-positive cells decreased with increasing drug concentrations, indicating that aNDR1 could effectively inhibit cell proliferation. A wound healing assay was used to assess cell migration, and the results showed that aNDR1 significantly inhibited the migration of prostate cancer cells in a dose-dependent manner (Figure 3B). Epithelial mesenchymal transformation (EMT) plays a critical role in tumor metastasis. Our previous study found that NDR1 could inhibit the metastasis of prostate cancer cells by inhibiting EMT progression (Yue et al., 2018). Therefore, we detected protein markers of EMT in DU145 cells by Western blotting. The results showed that the epithelial marker E-cadherin was obviously upregulated, and mesenchymal markers such as N-cadherin and vimentin were downregulated (Figure 3D), suggesting the negative regulation of aNDR1 toward NDR1-mediated EMT processing. NDR1 has been reported to promote apoptosis as a member of the Hippo pathway, which also participates in CRPC (Vichalkovski et al., 2008b). Therefore, we explored the effect of aNDR1 on apoptosis. After treatment with aNDR1 for 24 h, the apoptosis of PC3 and DU145 cells was measured by flow cytometry using an Annexin V-FITC apoptosis detection kit. We found that the proportion of apoptotic cells was significantly increased in a dose-dependent manner by aNDR1, and most of them were late apoptotic cells (Figure 3C). To further confirm the cell apoptosis induced by aNDR1, the expression levels of apoptosis-related proteins were measured. The expression of the antiapoptotic proteins Survivin, Bcl-2 and PARP was downregulated, and the proapoptotic proteins Bax and cleaved-PARP were upregulated (Figure 3D). In combination, these results indicated that aNDR1 had good inhibitory effects on tumor-related cellular processes, such as proliferation, migration, EMT and antiapoptosis, based on prostate cancer cell lines *in vitro*.

### 3.4 The antitumor activity of aNDR1 is NDR1-dependent

**FIGURE 4:**
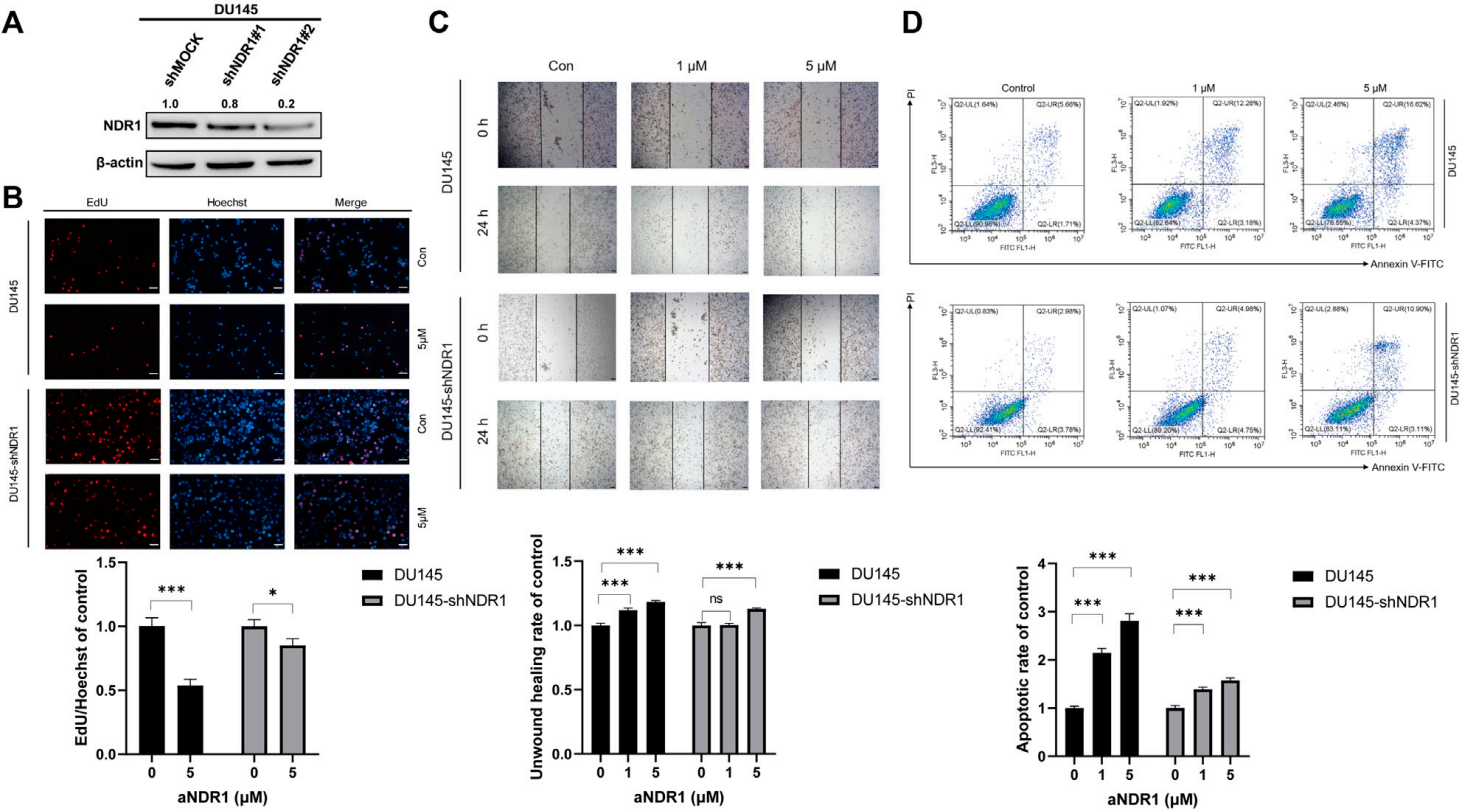
Knockdown of NDR1 prevents the anti-tumor activity of aNDR1. **(A)** NDR1 was knocked down in DU145 cells. **(B)** Knockdown of NDR1 prevented aNDR1-induced inhibition of proliferation. Scale bar, 50 μm. **(C)** Knockdown of NDR1 prevented aNDR1-induced inhibition of migration. Scale bar, 100 μm. **(D)** Knockdown of NDR1 prevented aNDR1-induced promotion of apoptosis. Data were represented as means ± SD of triplicate experiments. *, means *p* < 0.05, **, means *p* < 0.01, ***, means *p* < 0.001.

To confirm that the antitumor activity induced by aNDR1 specifically targets NDR1, we generated an NDR1-knockdown cell line named DU145-shNDR1, along with a control group DU145-shMOCK. The infection efficiency was assessed by Western blotting (Figure 4A). Then, we evaluated the effects of aNDR1 on DU145-shMOCK and DU145-shNDR1#2 cells, including EdU staining assays, wound healing assays and Annexin V-FITC apoptosis detection assays. As expected, knockdown of NDR1 in prostate cancer cells effectively prevented the inhibitory effects of aNDR1 on cell proliferation and migration, as well as the promotion of apoptosis (Figures 4B–D). These results demonstrate that the effects of aNDR1 on the phenotypes of cancer cells are dependent on the presence of NDR1, indicating the specificity of aNDR1 in targeting NDR1. These results further confirm that the observed anticancer effects of aNDR1 are mediated through its specific interaction with NDR1, highlighting its potential as a targeted therapeutic agent for prostate cancer.

### 3.5 aNDR1 increases the expression of NDR1 and can directly bind to NDR1

**FIGURE 5:**
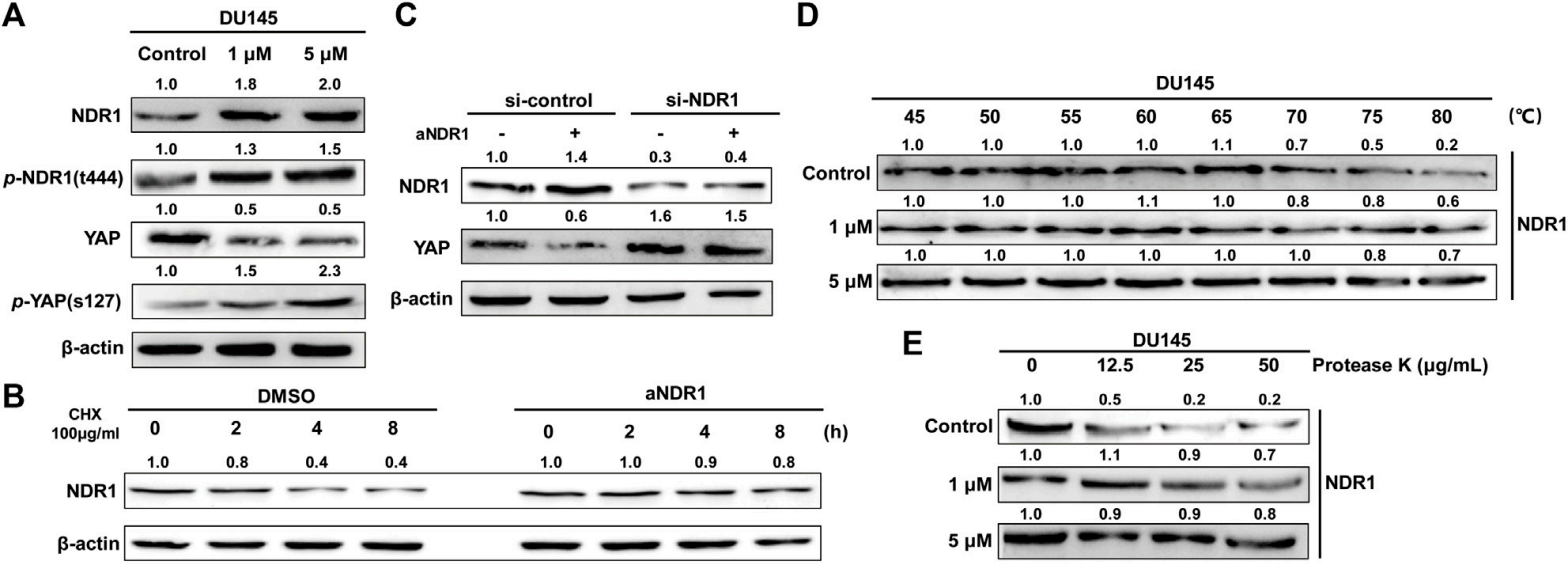
aNDR1 increases the expression of NDR1 and can directly bind to NDR1 **(A)** aNDR1 upregulated expression levels of NDR1 and affected its downstream proteins. The protein expression levels of NDR1, p-NDR1, YAP and p-YAP were detected by Western blotting after treatment with aNDR1 for 24 h **(B)** aNDR1 increased half-life of NDR1. DU145 cells were treated with CHX + DMSO or CHX + aNDR1 (5 μM) for 0, 2, 4, 8 h. **(C)** Protein levels of YAP, after 24 h of treatment with 5 μM aNDR1, in DU145 si-control and DU145 si-NDR1 group. **(D)** aNDR1 increased the thermal stability of NDR1 in CETSA assay. After treatment with DMSO or aNDR1 for 24 h, cells were heated at different temperatures for 5 min and then lysates were analyzed by Western blotting. **(E)** aNDR1 increased the anti-protease degradation ability of NDR1 in DARTS assay. DU145 cell lysates were incubated with DMSO or aNDR1 for 1 h at room temperature, followed by proteolysis with indicated ratios of protease K for 5 min, the lysates were analyzed by Western blotting.

NDR1 is a well-known member of the Hippo pathway, which can directly increase the phosphorylation of YAP and promote its degradation (Hergovich, 2013). Meanwhile, NDR1 was upregulated by MST1 as well as its phosphorylation of Thr-444 (Vichalkovski et al., 2008a). To explore whether aNDR1 directly targets NDR1, we examined the expression levels of NDR1 in DU145 cells treated with aNDR1. The results showed that the protein level of NDR1 was significantly upregulated (Figure 5A), which was related to NDR1 stabilization (Figure 5B). In fact, an enhancement of the NDR1 half-life by aNDR1 was observed upon the inhibition of protein synthesis with cycloheximide (CHX). Additionally, we found that YAP was downregulated and p-YAP was increased in the presence of aNDR1 (Figure 5A), and this effect was abolished by NDR1 small interfering RNA (siRNA) (Figure 5C).

Considering the favorable binding affinity between aNDR1 and NDR1 based on molecular docking prediction, we used the cellular thermal shift assay (CETSA) and drug affinity responsive target stability (DARTS) assay to confirm direct interaction, which have been widely applied in identifying target proteins of small molecule drugs (Park and Marqusee, 2005; Chang et al., 2012). The results showed that aNDR1 increased NDR1 thermal stability in a dose-dependent manner (Figure 5D). Meanwhile, aNDR1 partially protected NDR1 from protease-induced degradation (Figure 5E).

All these results suggested that NDR1 was the target protein of aNDR1, providing a promising lead compound for the treatment of prostate cancer.

### 3.6 aNDR1 inhibits the growth and metastasis of PCa cells *in vivo*

**FIGURE 6:**
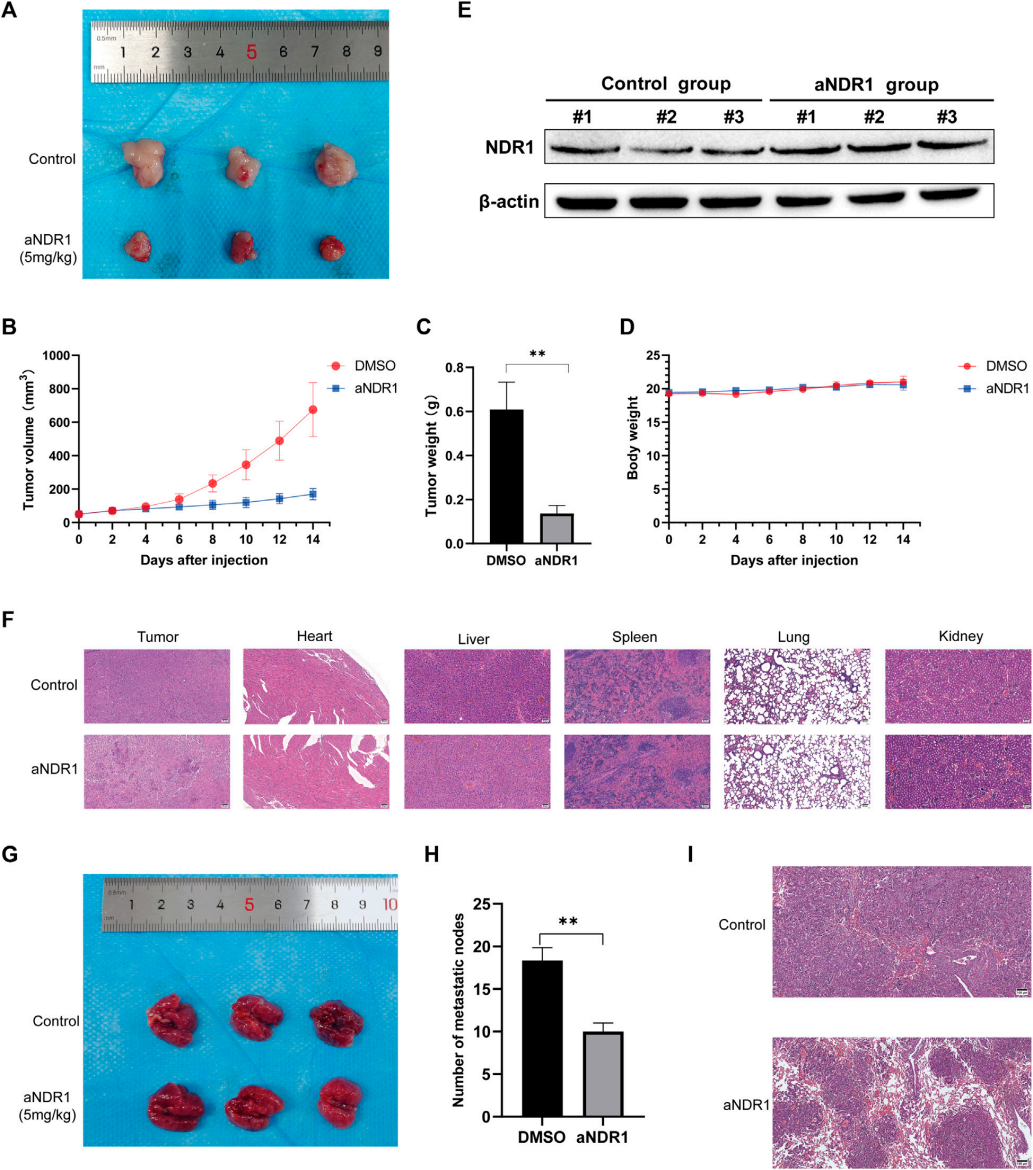
aNDR1 inhibits the growth and metastasis of PCa cells *in vivo* **(A)** The tumor image of control and aNDR1 treated group. **(B)** The tumor volume analysis between control and aNDR1 treated group. **(C)** The tumor weight analysis between control and aNDR1 treated group. **(D)** The mice body weight analysis between control and aNDR1 treated group. **(E)** Western blot analysis for NDR1 protein expression in tumor tissues from xenograft mice between control and aNDR1 treated group. **(F)** H&E staining of tumor, heart, liver, spleen, lung and kidney in control and aNDR1 treated group. Scale bar, 50 μm. **(G,H)** Effects of aNDR1 on tumor metastasis. **(I)** H&E staining of lung in mice metastasis models. Scale bar, 100 μm. Data were represented as means ± SD of triplicate experiments. *, means *p* < 0.05, **, means *p* < 0.01, ***, means *p* < 0.001.

To evaluate the antitumor effects of aNDR1 *in vivo*, a prostate cancer cell xenograft model was established by subcutaneously injecting DU145 cells into athymic nude mice. Tumor growth was monitored in nude mice that were intraperitoneally injected with aNDR1 (5 mg/kg) or DMSO every other day. As shown in Figures 6A–C, the volume and weight of the tumors were observably reduced in the aNDR1-treated group compared to those in the DMSO group. Importantly, no significant difference in body weight loss was observed between aNDR1-and DMSO-treated mice, indicating that aNDR1 had few toxic side effects on mice at our therapeutic concentration (Figure 6D). We also found that NDR1 expression levels in tumor tissues in the aNDR1-treated group were higher than those in the DMSO-treated group (Figure 6E). Consistently, the density of prostate cancer cells in tumor H&E staining was much lower than that in the DMSO group when treated with aNDR1 (Figure 6F). Furthermore, no obvious toxic pathologic damage in the heart, liver, spleen, lungs, or kidneys was observed by H&E staining (Figure 6F).

To investigate the effect of aNDR1 on tumor metastasis, we performed a tail vein injection of DU145 cells to induce lung metastasis. Visual observation (Figures 6G, H) and H&E staining of the lung sections (Figure 6I) revealed that aNDR1 effectively inhibited the metastasis of prostate cancer cells compared to that in the control group.

Overall, these results demonstrate that aNDR1 exhibits potent antitumor effects *in vivo*, including tumor growth and metastasis inhibition at the therapeutic concentration used, showing the potential of aNDR1 as a promising therapeutic agent for prostate cancer treatment.

## 4 Discussion

Androgen deprivation therapy is the first-line treatment for PCa, but most patients eventually develop resistance to ADT and progress into CRPC with a poor median survival rate of less than 2 years (Chandrasekar et al., 2015). Despite recent advances in diagnosis and treatment, CRPC treatment remains a significant medical challenge. Endocrinotherapy drugs such as enzalutamide and abiraterone and chemotherapy drugs such as docetaxel and cabazitaxel are the main treatment options for CRPC therapy, which greatly improves the prognosis in patients. However, inevitable drug resistance makes it difficult for these treatments to have long term benefits in patients (Cai et al., 2023). In addition, sipuleucel-T and radium-223 therapies have shown limited efficacy in advanced CRPC patients (Omlin et al., 2014). Current studies mainly focus on the development of new AR-targeted drugs, such as the AR N-terminal domain inhibitor QW07 (Peng et al., 2020), AR degrader HG122 (Cong et al., 2021), AR-V7 degrader nobiletin (Liu et al., 2021), and proteolysis-targeting chimera (PROTAC)-AR degrader ARV-110 (Wang et al., 2020). However, drug resistance of AR still remains an unsolvable problem, that may limit the clinical application of these drugs. Since the approval of the first small-molecule tyrosine kinase inhibitor (TKI), imatinib, for clinical use by the US Food and Drug Administration (FDA) in 2001 (Savage and Antman, 2002), a total of 89 anticancer small molecules have already been approved in the United States and China in 2021. With the development of elucidated mechanisms in CRPC, there have been some small-molecule drugs, such as olaparib and rucaparib, which target PARP, and ipatasertib, which targets AKT, in clinical treatment (Zhong et al., 2021c). These drugs have specific requirements for individual genes and mutations, which limits their application. The development of new therapeutic targets and drugs for advanced prostate cancer that do not depend on androgen signaling mechanisms is very important and urgently needed.

The Hippo pathway dysregulation promotes castration resistance and metastasis in prostate cancer, which is related to elevated activity of YAP (Le et al., 2022). Considering that YAP plays an oncogene role in a variety of tumors, several inhibitors of YAP have been developed, including verteporfin, Vestigial-like 4 and the 17-mer peptide, but have not been validated in prostate cancer cells (Salem and Hansen, 2019). NDR1 could phosphorylate YAP and promote its degradation in the cytoplasm in Hippo pathway (Watt et al., 2017; Koinis et al., 2022). Our previous study has reported that NDR1 inhibited the metastasis of prostate cancer cells by suppressing epithelial-mesenchymal transition (EMT), and decreased NDR1 expression might lead to a poorer prognosis (Yue et al., 2018). Therefore, increasing the activity of NDR1 may be a potential therapeutic treatment. We identified and characterized aNDR1 as a specific small-molecule lead compound as an agonist of NDR1 in this study. aNDR1 could effectively increase enzymatic activity and phosphorylation of NDR1, and we have demonstrated that aNDR1 directly bind to NDR1 through CESTA and DARTS experiments. We also found that aNDR1 upregulated expression levels of NDR1 by enhancing its half-life, and this further increased phosphorylation of YAP and promoted its degradation. At the same time, aNDR1 exhibited PCa cell-specific inhibition, while having no obvious effect on normal prostate cells. In future, the combination of NDR1 agonists and YAP inhibitors may have a better antitumor effect.

Uncontrolled tumor growth and metastasis are key contributors to cancer-related mortality. NDR1 has been reported to be involved in proliferation, apoptosis, and migration. Therefore, we examined the effects of aNDR1 on these aspects in prostate cancer cells. The results showed that aNDR1 significantly inhibited cell viability, proliferation and migration while promoting apoptosis in prostate cancer cells in an NDR1-dependent manner. Apoptosis, viability, and proliferation are inseparable processes that also influence each other. Of course, they also further influence cell migration. Interestingly, we found the effect of aNDR1 on apoptosis was the most significant. For the reason that in apoptosis detection experiments, we collect cells that have undergone late-stage apoptosis or even cell death in the supernatant. However, in other experiments, this effect and loss are unavoidable. To explore whether aNDR1 had satisfactory antitumor activity *in vivo*, xenotransplantation models and metastatic models were established. Compared with the control group, aNDR1 effectively inhibited subcutaneous tumors and lung metastatic nodules without causing abnormalities in body weight or affecting the heart, liver, spleen, lung, and kidney. Additionally, NDR1 also has been reported to play an antitumor role in colorectal cancer, T-cell lymphoma, glioblastoma. Although we did not investigate the effects of aNDR1 on these tumors. Given the conserved function of the NDR/LATS kinase family, aNDR1 may also exhibit enormous therapeutic potential.

EMT is associated with tumor progression, metastasis, and mediates resistance to conventional treatments and small-molecule targeted inhibitors. The general mechanism of EMT-related drug resistance is associated to increased drug efflux, slowed cell proliferation, and avoidance of apoptotic signaling pathways (De Las Rivas et al., 2021). Additionally, it was reported that castration could induce EMT that may enhance the stemness of cancer stem cells, which in turn results in castration-resistance and metastasis (Li et al., 2014). The ability of tumor cells to evade apoptosis also has been identified as one of the hallmarks of cancer. Dysregulation of the apoptotic pathway confers a survival advantage, enabling cancer cells to acquire drug resistance (Neophytou et al., 2021). As mentioned before, drug resistance of endocrinotherapy and chemotherapy make a poor prognosis of CRPC patients. Considering that aNDR1 effectively inhibited EMT progression and promoted apoptosis in CRPC cell lines, this may contribute to reducing drug resistance. aNDR1 in combination with endocrinotherapy drugs such as enzalutamide and abiraterone, chemotherapy drugs such as docetaxel and cabazitaxel would be promising treatments in the future.

However, we have not yet conducted pharmacokinetic experiments to determine the drug half-life and oral availability. Further improvements are needed to fully understand the *in vivo* efficacy of aNDR1. Additionally, modifying or allosterically targeting the chemical structure of aNDR1 may lead to the development of more potent small-molecule drugs.

Overall, the findings from this study highlight the potential of aNDR1 as a novel therapeutic lead compound for prostate cancer. Its specific targeting of prostate cancer cells, inhibition of tumor growth and metastasis, and favorable physicochemical properties make aNDR1 an exciting candidate for further preclinical and clinical investigations.

## Data Availability

The original contributions presented in the study are included in the article/Supplementary Materials, further inquiries can be directed to the corresponding authors.
