# Supplementary material for: Discovery of a small-molecule NDR1 agonist for prostate cancer therapy
Source: Front Pharmacol. 2024 Feb 12;15:1367358. doi: 10.3389/fphar.2024.1367358 (PMC10896269; doi:10.3389/fphar.2024.1367358)
Supplement: Supplementary file 1 [file Table1.docx]

**Supplementary Table 1：**ChEMBL analysis for potential small-molecule agonist of NDR1.


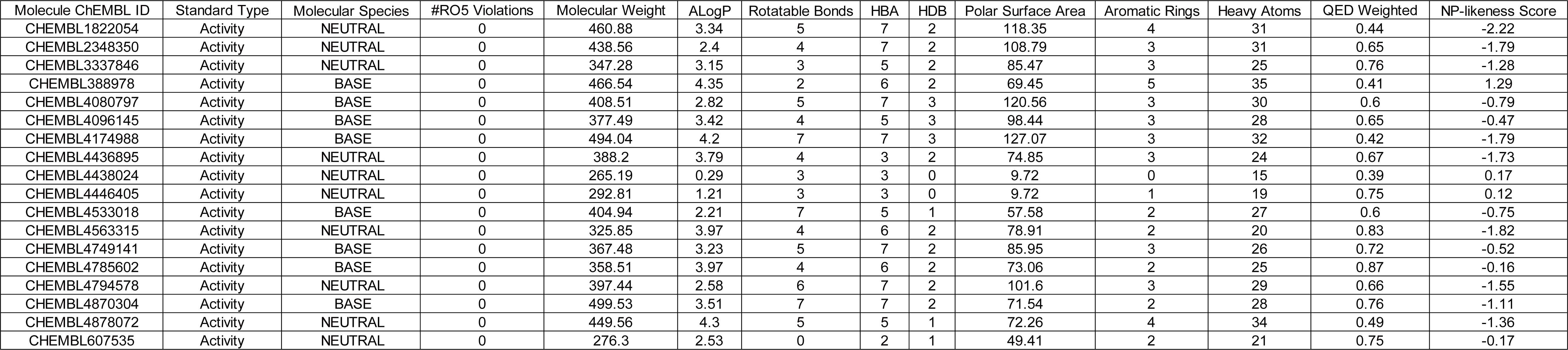


**
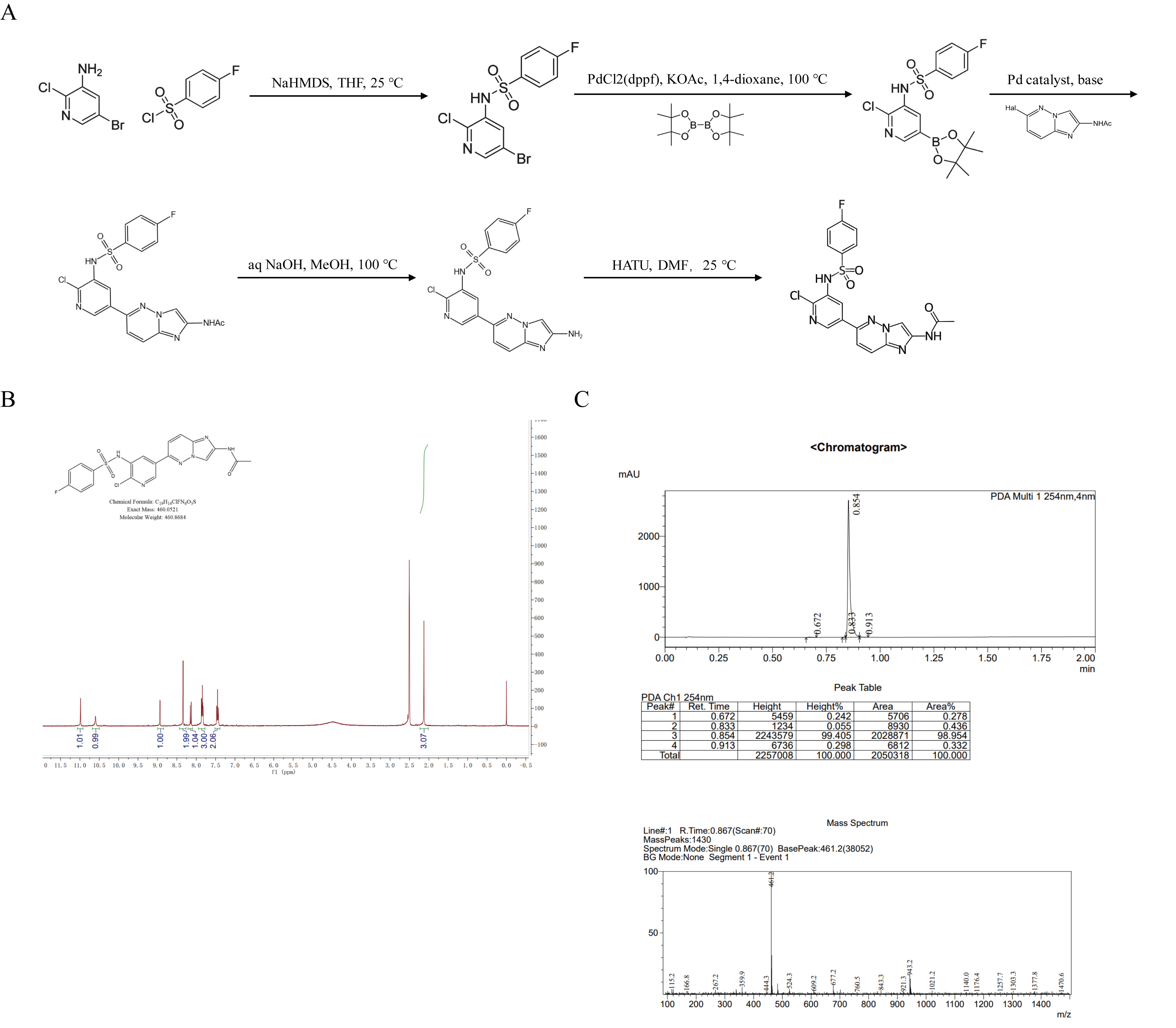
**

**Supplementary Figure 1. Synthesis and verification of aNDR1.**

(A) Schematic representation of chemical synthesis of aNDR1.

(B) ¹H NMR detection of aNDR1.

(C) LC-MS detection of aNDR1.


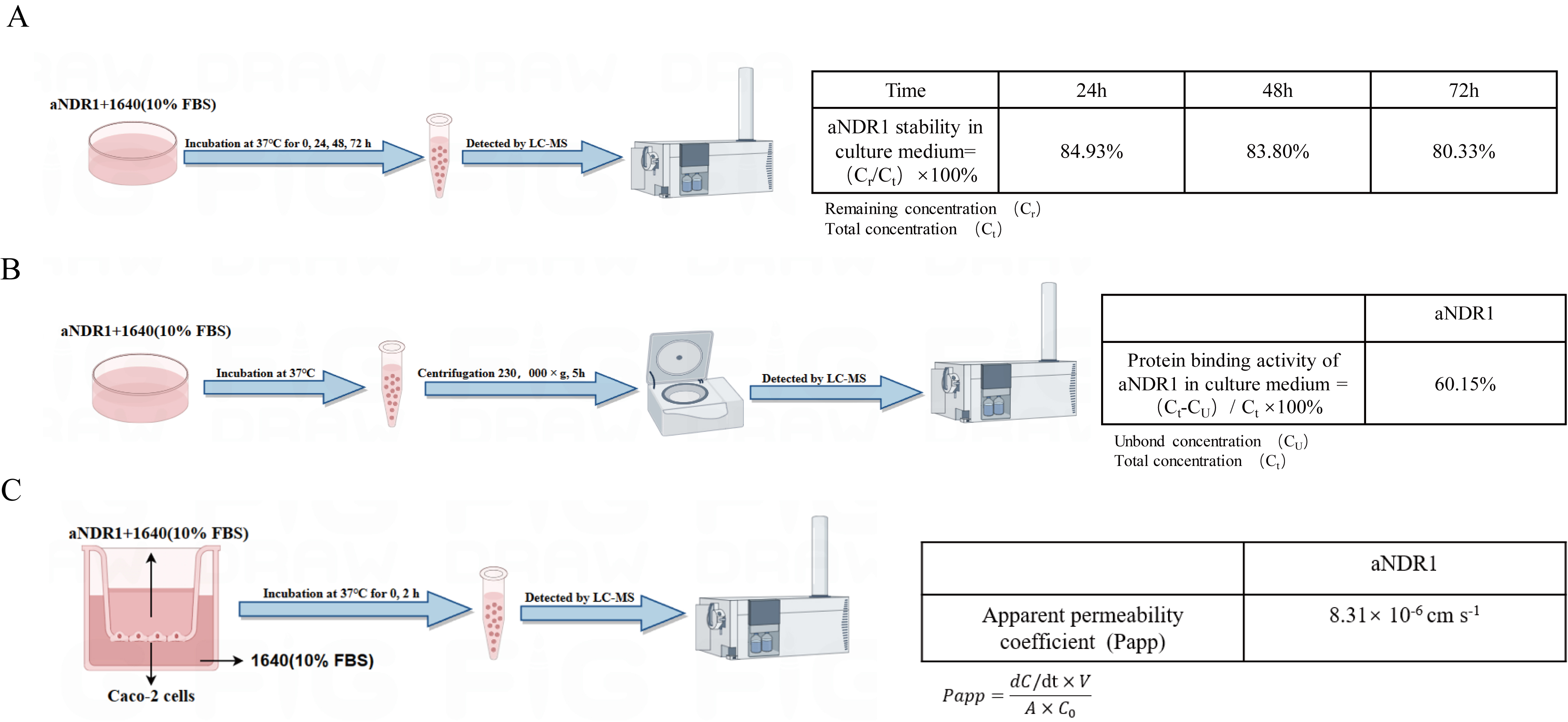


**Supplementary Figure 2. aNDR1 has a good physicochemical property.**

(A) The stability of aNDR1 in cell culture medium is shown.

(B) Protein binding activity of aNDR1 in cell culture medium is shown.

(C) apparent permeability coefficient of aNDR1 is shown.
